# Sexual Desire in Iranian Female University Students: Role of Marital Satisfaction and Sex Guilt

**Published:** 2014

**Authors:** Negar Teimourpour, Nahaleh Moshtagh Bidokhti, Abbas Pourshahbaz, Hadi Bahrami Ehsan

**Affiliations:** 1 PhD Student, Department of Clinical Psychology and Educational Sciences, University of Tehran, Tehran, Iran; 2 Associate Professor, Department of Clinical Psychology, University of Social Welfare and Rehabilitation, Tehran, Iran.; 3 Assistant Professor, Department of Clinical Psychology, University of Social Welfare and Rehabilitation, Tehran, Iran; 4 Associate Professor, Department of Clinical Psychology and Educational Sciences, University of Tehran, Tehran, Iran

**Keywords:** Female, Marital Satisfaction, Sex Guilt, Sexual Desire, Student

## Abstract

**Objective:**

To determine association between sexual desire and marital satisfaction and sex guilt among a sample of Iranian female university students.

**Methods:**

The data presented here were obtained from a total of 192 married Iranian female university students who were selected via a multi-cluster sampling method from universities of Social Welfare and Rehabilitation, Tarbiat Modarres, and Islamic Azad. The subjects' sociodemographic data, marital satisfaction (using ENRICH Marital Satisfaction Questionnaire), sex guilt (using Mosher Revised Sex-Guilt Inventory), and sexual desire (using Hurlbert Index of Sexual Desire) were gathered. Pearson correlation coefficient and regression analysis methods were used to analyse the data.

**Results:**

Findings showed there are significant relationships between sexual desire and marital satisfaction (r = 0.51, p < 0.01) and also between sexual desire and sex guilt (r = -0.44, p < 0.01). Also marital satisfaction and sex guilt were able to predict 31 percent of the variance of sexual desire.

**Conclusion:**

Marital satisfaction and sex guilt are two factors that significantly affect fluctuations in sexual desire of Iranian female university students.

## Introduction

Healthy sexual functioning is an important component of well-being. It is able to produce a mutual and joint pleasure in couples and even help them cope more efficiently with stresses and problems of daily life (1). An important factor in a happy and successful marriage is having a pleasurable sex life, while non-pleasurable sex life can lead to frustration and feeling of insecurity in couples (1). Dysfunctional sexual relationships may occur due to different reasons, but sexual desire is among the main factors that lead to difficulty in sexual functioning. 

In the US, 43% of women and 31% of men suffer from some kind of sexual difficulty and 22% of women and 5% of men experience problems related to low sexual desire in their life span (2). According to statistics, sexual desire problems -especially low sexual desire- is the most common sexual complaint among women (3, 4). This is a complicated and interesting phenomenon which has puzzled the scholars for many years. Various definitions of sexual desire have been presented, for example Levine’s multidimensional model of sexual desire. He proposes that sexual desire is the force that makes people behave in a sexual manner and has three components: (1) “sexual drive”, the biological component which is mediated by the neuroendocrine system; (2) “sexual wish”, the social component representing societal expectations; (3) “sexual motive”, individual/interpersonal component reflecting a person's tendency to engage in sex with a particular partner. He also mentions that the spectrum of sexual desire intensity fluctuates between aversion, disinclination, indifference, interest, need, and passion. Although many individuals have a pattern of desire throughout their lives, an individual's sexual desire may vary along this spectrum (5). Regan and Atkins define sexual desire as a motivational state that leads to interest and inclination to a sexual object or sexual activity. They distinguish sexual desire from psychological and physiological sexual arousal (6).

Different factors affect the fluctuation and changes in sexual desire which can be categorized into physiological factors and psychological factors (7). Different types of cancer as well as diabetes mellitus are examples of physiological risk factors. Emotions, maladaptive cognitions, lack of education regarding sexual functioning, and couple distress are among examples of psychological risk factors (7). Also, cultural factors such as racial, ethnic and religious background often affect a person's beliefs, expectations, and behaviors in sexual relationships (7). It has been reported that cognitive factors (mainly automatic thoughts during sexual activity) were the best predictors of sexual desire. In a more specific way, age, failure/disengagement thoughts, and lack of erotic thoughts during sexual activity, showed a significant direct effect on reduced sexual desire. Furthermore, sexual conservatism beliefs and medical factors showed indirect effects, acting on sexual desire via the lack of erotic thoughts, and failure/disengagement sexual thoughts, respectively (8).

There are so many different psychological factors that may affect sexual desire which have not been studied yet in Iranian population. One of these factors is marital satisfaction. Marital satisfaction refers to an individual’s experience in marriage that can only be assessed by each person's response to the degree of pleasure obtained from marriage (9). Studies have found direct relationship between frequency of sexual intercourse and marital satisfaction (10, 11). In other words, men and women who reported that they are happy and satisfied with the frequency of their sexual activity had higher levels of sexual and marital satisfaction. Also relationship between marital satisfaction and sexual satisfaction has been confirmed in various studies (12-14). In another study, Hurlbert et al. found that communication between couples is an important factor that affects sexual desire and mental functioning of women with hypoactive sexual desire disorder (15).

The lack of research investigating sexuality within marriage is, by itself, a compelling reason to explore this topic. In addition, since marriage plays a vital role in the Iranians’ life and on the other hand, sexuality issues are usually unspoken in Iran, so that examining the association of sexual desire with marital satisfaction seems necessary. 

Feeling guilty about sexual issues is another factor that may affect sexual desire (16). Sex guilt affects different aspects of personal life, especially relationship with partner. People who feel guilty always worry about violating moral rules and they feel like they are bad persons when they engage in a sexual behavior. Attitude toward sexual issues, such as sex guilt, is one of the most common reasons of sexual dissatisfaction and sometimes even formation of sexual dysfunction. These kinds of attitudes lead to problems such as premature ejaculation and sexual impotence in men and low sexual desire and anorgasmia in women (16). Because sex guilt has emotional and personality components, it influences not only the individual's perception of sexual behavior, but also can aid or inhibit the causation of specific sexual behaviors. Thus sex guilt influence the continuation of sexual behavior (more sex guilt leads to lesser chance of repeating of the behavior that evokes sex guilt) and may have baneful effect on future sexual function and satisfaction (17). Also research has shown that discomfort with sexual issues and sexual dissatisfaction (psychological and physiological) are correlated with high levels of sex guilt during first intercourse, high current and future sex guilt, and increased chance of sexual dysfunction (17). Mehrabi and Dadfar also found that sex guilt is one of the factors that influence sexual dysfunction (18). There has been no study in the field of the relationship between sex guilt and sexual desire in Iranian literature, so this factor was also selected to be investigated here.

Considering the issues presented above, it seems that despite high rate of sexual desire problems in women and its negative consequences, there are still so many questions that have not been answered in this area, especially in Iranian women. This is the reason why in this study the relationship between marital satisfaction and sex guilt with sexual desire is being addressed. 

We hypothesized that marital satisfaction is positively related to sexual desire and sex guilt is negatively related to sexual desire in Iranian women. We also intended todetermine the role of each of these variables in explaining the variations and fluctuations of sexual desire in women.

## Materials and Methods

Study population consisted of all married female students aged 18-40 years who were selected via a multi-cluster sampling method from Social Welfare and Rehabilitation, Tarbiat Modarres, and Islamic Azad Universities [North branch in Tehran] in undergraduate and graduate programs in 2010. Exclusion criteria were pregnancy, menopause, having a specific disease such as diabetes mellitus, thyroid diseases, any type of cancer, central nervous system diseases (e.g., multiple sclerosis), psychological disorders, consumption of a specific drug (such as sexual desire stimulant or reducer and psycho-active drugs), consumption of alcohol and narcotics, history of divorce, and the students of Psychology and Counseling (because of familiarity with the variables and questionnaires).

Data gathering was carried out via pre-designed questionnaires and checklists (see below) during the last 30 minutes of class time. First, the researcher gave an explanation about the questionnaires, the topic of the study, the inclusion and exclusion criteria. Then, the eligible students were asked to complete the questionnaires. Confidentiality was preserved and the participants were not asked to write down their names on the questionnaires. All the participants completed consent forms before participation. While the sample population was 200 subjects, eventually data of 192 women were used (three questionnaires were excluded because of invalid answering, 2 were excluded because of filling out the questionnaires incompletely and 3 were excluded because of being student in the major of psychology).

***Measures***

*Demographic questionnaire*

A questionnaire was designed by the researchers to assess demographic data including sex, age, marital status, marriage duration, education, university, pregnancy status, consumption of a specific drug, alcohol and narcotic, and any physical disease or psychological disorder.

*ENRICH marital satisfaction questionnaire*

This questionnaire was originally designed by Foweres and Olson and the original form has 112 questions (19). In this study the short form (47 questions) was implemented. Psychometric properties of the questionnaire are satisfactory (19). Cronbach’s alpha of the short form (Persian version) was 0.95 in Soleimanian' study (20). Correlation of the questionnaire with life satisfaction was 0.32-0.41 which indicates good construct validity. Also the scale can discriminate between couples with marital problems and those without (20).

*Revised Mosher sex guilt inventory*

This inventory consists of 50 questions and has been designed by Mosher in 1998 in order to assess guilt about sexual issues. It is in Likert scale (0-6) and measures sex guilt in a range of 0-300. Psychometric properties of this scale have been confirmed in several studies (21). In Iran, the Cronbach’s alpha of the questionnaire among 917 university student was 0.87. Test-retest reliability (with two weeks interval) among 225 university student was 0.77. Content validity was assessed using Kendal coefficient among 7 psychology professors and was 0.82 which is significant. Convergent and discriminate validity of the questionnaire was also confirmed (22).

*Hurlbert Index of sexual desire (HISD)*

The HISD was invented by Apt and Hurlbert and consists of 25 questions in Likert scale (0-4) with the total score range of 0-100. Cronbach’s alpha of the questionnaire and test-retest reliability (with two weeks interval) was 0.95 and 0.86 (23). Psychometric properties of the questionnaire are satisfactory (23). Before implementation, the index was translated by the researcher. Afterwards, a person who was completely fluent in both English and Persian language but was unfamiliar with the subject of the study translated the index back to English. Then items which were not compatible with the original questions were revised. At this stage the questionnaire was completed by 10 subjects while researcher asked them to read the questions one by one and tell the meaning of each item to make sure that it is comprehensible in Iranian subjects. Items which implied a different meaning were revised. Then questionnaire was completed by 45 subjects who met the inclusion criteria but were not among the study sample. The Cronbach’s alpha was 0.89 and test-retest reliability (with two weeks interval) was 0.89. Upon completion of the study, the Cronbach’s alpha (among 192 women) was 0.93. Content validity was assessed using Kendal coefficient among 4 psychology professors and was 0.82, which is satisfactory.

***Data analysis***

Descriptive statistics were used to calculate frequency, mean, standard deviation (±SD), absolute ranges and subject's demographic characteristics. Pearson correlation was applied in order to determine correlations between variables of the study. Stepwise regression analysis was done to determine the share of marital satisfaction and sex guilt in explaining the variance of sexual desire in women. Data analysis was carried out using SPSS for Windows version 16.0 (SPSS, Inc., Chicago, IL, USA). 

## Results

The age range of the participants was between 18-40 years with a mean (±SD) age of 26 (±4) years. Range of marital duration was between 2-150 months with a mean (±SD) of 41 (±36) months. Sixty-seven participants were studying in the Bachelor Degree, 102 were in Masters Degree, and 23 were at PhD level. Eighty-two participants were student in University of Social Welfare and Rehabilitation, 67 were in Tarbiat Modarres University and 43 in Islamic Azad University. 

Scores of ENRICH marital satisfaction were between 87-224 with a mean (±SD) of 167 (±30). Scores of Mosher Sex guilt inventory were between 24-270 with a mean (±SD) of 160 (±50). Scores of Hurlbert index of sexual desire were between 15-91 with a mean (±SD) of 60 (±17).

To examine the hypotheses of the study, Pearson correlation coefficients between the scores of marital satisfaction, sex guilt and sexual desire were calculated. There were significant relationships between sexual desire and marital satisfaction (r = 0.51, p < 0.010) and sex guilt (r = -0.44, p < 0.010). Correlations among all variables of the study and descriptive information of scales are presented in table 1.

**Table 1:** Results of bivariate correlation analyses among all variables of the study in 192 Iranian female university students

	**Sexual desire**	**Marital satisfaction**	**Sex guilt**
**Sexual desire**	1		
**Marital satisfaction**	0.51*	1	
**Sex guilt**	-0.44*	-0.45*	1
**M** **ean ** **±** ** SD** †	62 ± 16	167 ± 34.35	163.36 ± 57
**Absolute rage**	15-87	87-224	24-270

* p < 0.010;

† Standard deviation

To determine the share of marital satisfaction and sex guilt in explaining the variance of sexual desire in women, regression analysis method was used. The results are shown in table 2.

**Table 2:** Stepwise multiple regression results for marital satisfaction and sex guilt, predicting sexual desire in 192 Iranian female students

	**Adjusted R** ^2^		**F change**	**Β**	**T**	**p**
**Marital**		025†	67.6	0.51	8.2	0.001
**Marital, guilt**		0.31‡	44.1	0.39 -0.26	5.8 -3.9	0.001

† Predictors: (constant), marital satisfaction

‡ Predictors: (constant), marital satisfaction, sex guilt

As it is shown, adjusted R^2^ in the last step is 0.31 (p < 0.001), which means that marital satisfaction and sex guilt can explain 31% of the variance of sexual desire.

## Discussion

As it was mentioned earlier, there is a significant positive relationship between marital satisfaction and sexual desire. This is in line with previous research on the relationship between marital and sexual satisfaction with frequency of intercourse (10, 11) and the relationship between marital and sexual satisfaction (12-14). Also Carvalho and Nobre in their study found that participants from the low desire group presented less dyadic adjustment (24). Overall we can propose that marital satisfaction is one of the most important factors affecting sexual desire. It seems that conflict between partners leads to decrease in sexual satisfaction, frequency of intercourse, and lack of sexual interest and desire (24). But we should be aware that the sample of this study was only women. Marital dissatisfaction may have different influences on sexual desire among males. So, choosing couple samples and comparing their results with each other can lead to interesting results.

It was confirmed that there is a significant negative relationship between sex guilt and desire. Person's attitude toward sexual issues, such as sex guilt, is one of the most common reasons of formation of sexual dissatisfaction and problems. These kinds of attitudes lead to problems such as premature ejaculation and sexual impotence in men and low sexual desire and anorgasmia in women (16). Sadock and Sadock also believed that sex guilt and moral restrictions influence sexual dysfunction, especially low sexual desire and arousal disorder and anorgasmia (25). We can suppose that sex guilt is a cultural issue. In eastern cultures- especially Islamic cultures- sexual issues are taboo and people rarely discuss about sexual subject in public places or educational institutes. On the other hand sexual intercourse before marriage is prohibited. These restrictions are tougher on girls in Iran because they have to be virgins till they get married. Girls repeatedly receive this message that sexual intercourse and issues are inappropriate and sinful. As a result this guilt toward sexual issues establishes and they feel guilty when they have sex even with their husbands. This is more prominent when years between puberty and marriage gets longer as individual has to struggle with that sex guilt for a longer time and on a daily basis and sex basically becomes conflicting for them.‬

The limitations of this study and recommendations for future studies are listed below:

1. Sample of the study was only women. The results may be different in men. So, choosing couple samples and comparing their results may lead to interesting findings.

2. The age range of the sample was between 18 and 40 years, so choosing women of different age groups is suggested.

3. Only students were studied here. Choosing a sample of general population is suggested. 

4. Self report measures have their own limitations, so using qualitative methods (e.g. interview) is recommended for further studies.

## Authors' contributions

NT and NMB conceived and designed the evaluation and helped to draft the manuscript. AP participated in designing the evaluation. NT collected the clinical data. NMB re-evaluated the clinical data. NT and NMB interpreted them. All authors participated in statistical analysis. BE revised the manuscript. All authors read and approved the final manuscript.

## Notes

**Declaration of interest: **This study was based on the thesis of the first author to get her Masters Degree in Clinical Psychology, University of Social Welfare and Rehabilitation, Tehran, Iran.
